# Supplementary material for: Which National Factors Are Most Influential in the Spread of COVID-19?
Source: Int J Environ Res Public Health. 2021 Jul 16;18(14):7592. doi: 10.3390/ijerph18147592 (PMC8307075; doi:10.3390/ijerph18147592)
Supplement: Supplementary file 1 [file ijerph-18-07592-s001.zip › ijerph-1256444-supplementary.pdf]

International Journal of
Environmental Research and Public Health

MDPI

Supplementary Information- Which National Factors are Most Influential in the Spread of COVID-19?

Table S1. List of the national factors used.

Summary of national factors			
**Source**	**Category**	**Variable name**	**Variable information**
Our World in Data	*Territory, Population*	Population (2020)	Population in 2020
		Population Density (2020)	The number of people in a given area divided by the area of the given area. Usually expressed as population per km^2
	National Accounts	GDP per capita	Gross domestic product divided by the number of people
	Age	Median Age (2020)	Median age of the population, UN projection for 2020
		Aged 65 older	Share of the population that is 65 years and older, most recent year available
		Age 70 older (2015)	Share of the population that is 70 years and older in 2015
		Life Expectancy (2019)	The average number of years a 0-year-old is expected to survive in the future
	Health, Society, And Welfare	Cardiovascular Death Rate (2017)	Death rate from cardiovascular disease in 2017 (annual number of deaths per 100,000 people)
		Diabetes Prevalence (2017)	Diabetes prevalence (% of population aged 20 to 79) in 2017
		Female Smokers	Share of women who smoke, most recent year available
		Male Smokers	Share of men who smoke, most recent year available
		Handwashing Facilities	Share of the population with basic handwashing facilities on premises, most recent year available
		Hospital Beds per thousand	Hospital beds per 1,000 people, most recent year available since 2010
		Extreme Poverty	Share of the population living in extreme poverty, most recent year available since 2010
	Education, Culture, Science	Human Development Index	Summary measure of average achievement in key dimensions of human development: a long and healthy

			life, being knowledgeable and have a decent standard of living
KOSIS	*Trade*	Exports to GDP Ratio (2016)	
		Imports to GDP Ratio (2016)	
	Environment	Average Annual Temperature (1961-1990)	30-year statistics from 1961 to 1990 based on observed temperature
		Annual Precipitation (1961-1990)	30-year statistics from 1961 to 1990 based on observed precipitation
	National Accounts	Gross Domestic Product (2018)	A monetary measure of the market value of all the final goods and services produced in a specific time period
	Territory, Population	Proportion of Urban Population (2018)	The proportion of the population living in urban areas among the total population (However, the concept of cities in each country is different, and the urban population of each country is based on the census of each country.)
		Total Fertility Rate (2020)	Average of the number of children a woman has in her lifetime
		Net Migration Rate (2020)	The number of the number of transferees minus the number of transferees over a specific period divided by the total population of the period
	Education, Culture, Science	Number of international travelers (2017)	The number of foreign tourists with each country's nationality expressed in units of thousands
		Number of foreign visitors (2017)	The number of foreign visitors to each country expressed in units of thousands
		National Competitiveness (2019)	The ranking of national competitiveness announced by the Swiss Institute of International Business Development (IMD) (Consider areas such as economic performance, government efficiency, corporate efficiency, and infrastructure)
	Health, Society, And Welfare	Infant vaccination rate (2017)	The infant immunization rate is the percentage of infants 12 to 23 months of age who were vaccinated 12 months before or before the

		investigation of four diseases, measles and DPT (diphtheria, whooping cough, tetanus)
	Public Social Welfare Expenditure (2016)	Social benefits or financial assistance by public institutions while households or individuals are in a disadvantaged environment (Expressed as a percentage of GDP)
	Pharmaceutical Sales (2018)	Total sales based on the retail price of the drug's final sales, classified by ATC code (Anatomical Therapeutic Chemical Classification System)
	Percentage of malnourished population (2016)	Percentage of the population who consistently consume food at a level lower than the minimum dietary energy consumption
Age	Aging index (2020)	(Population over the age of 65 in the current year ÷ Population between 0 and 14 in the current year) × 100

Pseudo-code of Segmentation Algorithm

Here, we denote $\hat{f}(t)$ by Y_t.

Algorithm 1 Peak Detection Algorithm

Input: Y_i $where$ $i \in \{1, 2, ..., t_{max}\}$
Output: I

1. (Initialization)
declare $t = 4, \mathrm{I} = \emptyset, c = 0.1\ argmax_{i \in T}|\Delta^2 Y_i|$ where $T = \{1, 2, ..., t_{max} - 2\}$;

2. (Local maximum condition)
while $t = t_{max} - 4$ **do**
if $\Delta Y_t \cdot \Delta Y_{t+1} \leq 0, \Delta^2 Y_t \leq -c$ **then**
$I \leftarrow I \cup \{t\}$; $t \leftarrow t + 1$;
end if
end while

3. (Excluding small peaks)
if $\mathrm{I} = \emptyset$ **then**
$\mathrm{I} \leftarrow \mathrm{I} - \{i | i \in \mathrm{I}, Y_i \leq 0.2 Y_{max}\}$;
end if

4. (Resolution criteria)
if $n(\mathrm{I}) < 2$ **then**
declare $p = 1$, $k = 1$, $A = \{i | I_k \leq i \leq I_{k+1}\}$ $where\ I_j\ is\ the\ j - th\ smallest\ integer\ in$ I;
while $p \leq n(\mathrm{I}) - 1$ **do**
if $argmax_{i \in A} Y_i \geq 0.8 max\{Y_k, Y_{k+1}\}$ **then**
if $Y_k \leq Y_{k+1}$ **then**
$\mathrm{I} \leftarrow \mathrm{I} - \{I_k\}$;
else
$I \leftarrow \mathrm{I} - \{I_{k+1}\}$;
end if

else
$k \leftarrow k + 1$
end if
$p \leftarrow p+1$
end if

5. (Exclude peaks which are vibrations on increasing trend)
if $n(\text{I}) \geq 1$ **then**
declare $z = I_{n(I)}, L = \{i | z \leq i \leq min\{z + 30, t_{max}\}\};$
if $z \leq argmin_{i \in L} Y_i$ **then**
$I \leftarrow \text{I} - \{z\}$
end if
end if

Algorithm 2 Breakpoint Detection Algorithm

Input: Y_i $where$ $i \in \{1, 2, \dots, t_{max}\}$
Output: B

1. (Initialization)
declare $\text{B} = \emptyset, c = 0.1\ max(|\Delta Y_t|);$

2. (Between peaks)
if $n(\text{I}) \geq 2$ **then**
for each $j \in \{1, 2, \dots, (n(\text{I}) - 1)\}$ **do**
$\text{B} \leftarrow \text{B} \cup \{argmin_i Y_i | i = (\text{I}_j + 1), \dots, \text{I}_{j+1})\};$
end do
end if

3.(After the last peak)
declare $\text{U} = \{i \in \{(\text{I}_{n(I)} + 1), \dots, t_{max}\} | \Delta Y_i \geq c\};$
if $n(\text{I}) \geq 1, n(U) \geq 5$ **then**
$\text{B} \leftarrow \text{B} \cup \{argmin_i Y_i | i = (\text{I}_{n(I)} + 1), \dots, t_{max}\};$
end if

Figure S1. Pseudo-codes for the Segmentation Algorithm.

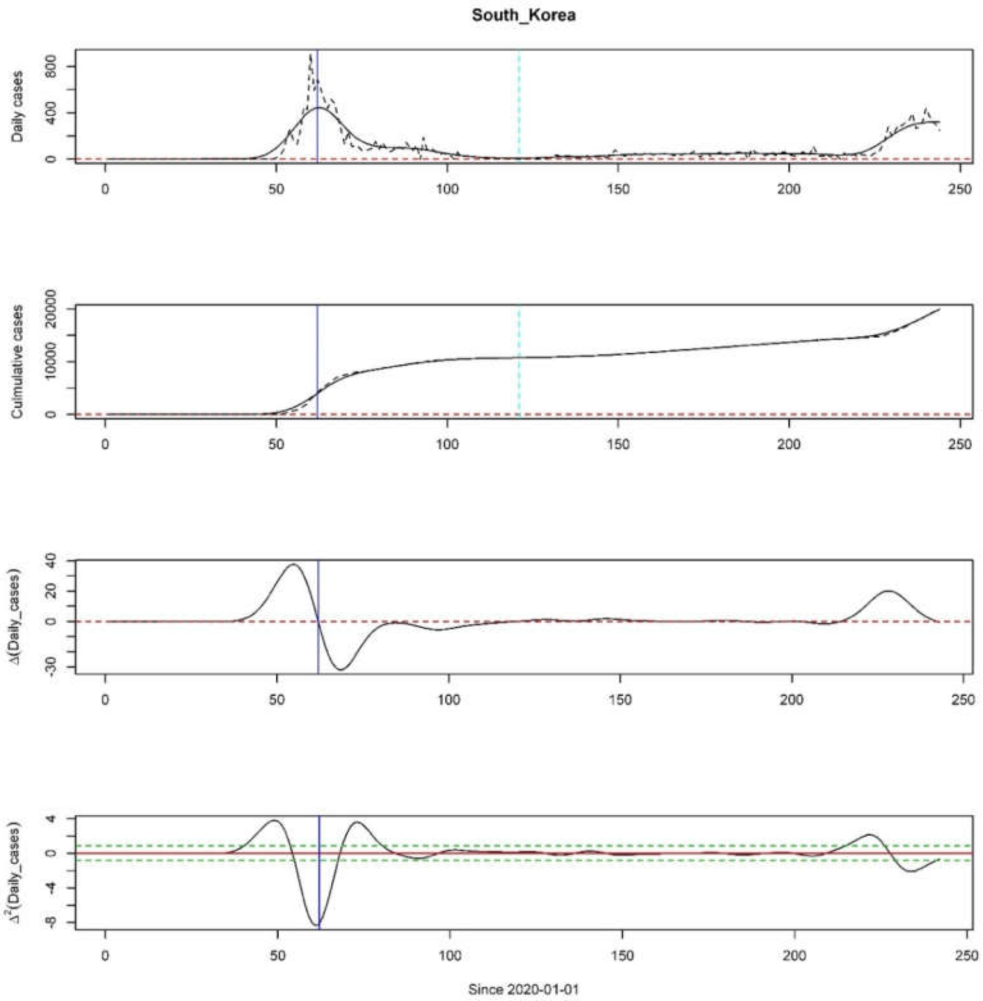

Figure S2. Segmentation algorithm applied to South Korea's COVID-19 daily new confirmed cases. The blue line represents the peak and dotted sky-blue line represents breakpoint. In the 1st plot, black solid line represents $\hat{f}(t)$ and black dotted line represents Y_t. The 2nd plot represents cumulative confirmed cases of Y_t(black dotted line), $\hat{f}(t)$(black solid line). 3rd, 4th plots are graphs of $\Delta\hat{f}(t), \Delta^2\hat{f}(t)$. In 4th plot, green dotted line represents sensitivity level. If $\Delta^2\hat{f}(t)$ is above the upper green dotted line, $\hat{f}(t)$ is concave. On the other hand, if $\Delta^2\hat{f}(t)$ is below the lower green dotted line, $\hat{f}(t)$ is convex.

Table S2. Parameter values estimated from segmented Logistic model. α is the maximum number of predicted cumulative confirmed cases, β is the time when we start to see a rise in the number of confirmed cases, γ is the increase rate of the number of confirmed cases. NA indicates no values returned due to failure of the model to converge.

Segmented Logistic Model								
		1st Segment			2nd Segment			
**Country**	**Maximum Cumulative cases**	α	β	γ	α	β	γ	**MSSE**
Afghanistan	38162	37269.8509	5.6770	0.0743	NA	NA	NA	0.0285
Albania	9380	29521.2462	5.3078	0.0268	NA	NA	NA	0.0292
Algeria	44146	12006.8756	3.7504	0.0622	34805.8852	3.8383	0.0758	0.0296
Angola	2624	3210.7931	5.3484	0.0605	NA	NA	NA	0.1101
Argentina	401226	648605.7127	7.0822	0.0434	NA	NA	NA	0.0253
Armenia	43750	43766.2039	5.5276	0.0542	NA	NA	NA	0.0164
Aruba	1997	2036.3519	23.7171	0.1651	NA	NA	NA	0.4136
Australia	25670	6874.2219	5.5232	0.1993	19130.8848	5.7873	0.1019	0.0723

Austria	27218	15908.5183	4.2740	0.1596	16850.1551	3.8620	0.0498	0.1512
Azerbaijan	36309	39349.2968	5.7054	0.0523	2314.9887	3.1543	0.3069	0.0355
Bahamas	2167	2725.7862	11.5786	0.0902	NA	NA	NA	0.1529
Bahrain	51574	52482.2417	5.2532	0.0432	NA	NA	NA	0.0522
Bangladesh	310822	309918.2068	4.8411	0.0488	NA	NA	NA	0.0632
Belarus	71687	67674.9628	4.3724	0.0640	3143.6539	3.1136	0.2155	0.0513
Belgium	85442	59026.3230	4.1785	0.1002	25594.5256	4.4737	0.0989	0.0589
Bolivia	115968	136119.5696	5.7989	0.0465	NA	NA	NA	0.0150
Bosnia and Herzegovina	19789	NA	NA	NA	NA	NA	NA	NA
Brazil	3862311	4453662.2090	5.5266	0.0408	NA	NA	NA	0.0335
Bulgaria	16190	2847.4247	3.4207	0.0696	14226.6135	4.0922	0.0691	0.0347
Cameroon	19142	21027.7178	4.5991	0.0509	6228.8127	2.3642	0.1062	0.2697
Canada	127940	102198.2467	4.1626	0.0711	23205.5724	2.7602	0.0880	0.0676
Cape Verde	3852	451.6892	3.0462	0.1095	3709.0145	3.4858	0.0529	0.0886
Central African Republic	4700	4681.4703	4.6191	0.0867	NA	NA	NA	0.0584
Chile	409974	386213.7660	6.2397	0.0635	NA	NA	NA	0.1951
China	89868	84438.2778	5.2721	0.1958	NA	NA	NA	0.3252
Colombia	607938	1028559.6439	7.1185	0.0441	NA	NA	NA	0.0114
Congo	3979	4512.6300	5.1438	0.0508	NA	NA	NA	0.2155
Costa Rica	39699	53094.5266	8.5012	0.0555	NA	NA	NA	0.0682
Cote' d Ivoire	17948	19182.4988	5.3947	0.0525	889.9903	3.0251	0.3372	0.0898
Croatia	10123	2171.0152	3.1237	0.1317	13184.8289	4.3545	0.0482	0.2648
Cuba	3973	1856.7584	3.4571	0.1238	424.2954	3.0161	0.1868	0.0354
Czechia	24367	8425.2168	3.4373	0.1147	20390.7458	3.6332	0.0476	0.1193
Democratic Republic of the Congo	10044	9704.1760	4.8552	0.0575	208.7552	3.3273	0.6117	0.0521
Denmark	16700	12229.4185	3.0248	0.0747	4665.2456	4.2840	0.1044	0.1107
Djibouti	5385	1051.5098	4.9399	0.3115	3980.2177	4.0720	0.1357	0.2622
Dominican Republic	94241	143968.8886	4.5142	0.0321	NA	NA	NA	0.0887
Ecuador	117639	41796.9588	5.4738	0.1175	84434.3146	3.0774	0.0511	0.2161
Egypt	98727	99949.4036	6.4644	0.0618	NA	NA	NA	0.0104
El Salvador	25729	34877.7380	5.4834	0.0438	NA	NA	NA	0.0450
Equatorial Guinea	4941	1692.8579	3.5291	0.0881	1949.5310	5.0940	0.2163	0.6387
Estonia	2373	1856.0023	2.6937	0.1019	534.2583	4.6425	0.0788	0.2039
Eswatini	4561	6353.9951	5.1505	0.0475	NA	NA	NA	0.0585
Ethiopia	51122	NA	NA	NA	NA	NA	NA	NA
Finland	8077	7134.7878	3.2117	0.0755	1033.3456	4.0422	0.0980	0.0628
France	278709	147092.5435	4.6031	0.1069	NA	NA	NA	0.1265
Gabon	8505	3546.9607	5.1536	0.1172	2908.7589	3.0856	0.1821	0.0465
Gambia	2963	3105.6876	6.4323	0.1387	NA	NA	NA	0.0751
Georgia	1487	175285.9159	4.7540	0.1159	152925.9558	3.6806	0.0373	0.0995
Germany	242381	9712.5829	5.4931	0.1023	41388.5308	3.5723	0.0616	0.1187
Greece	10134	2731.1379	3.3367	0.1120	NA	NA	NA	0.0929
Guam	1347	NA	NA	NA	NA	NA	NA	NA
Guatemala	73912	80489.4110	6.0005	0.0525	NA	NA	NA	0.0586

Guinea	9371	5303.4292	3.3438	0.0680	4510.1775	2.8192	0.0736	0.0819
Haiti	8209	7622.6502	4.7753	0.0824	NA	NA	NA	0.1544
Honduras	60174	56907.2114	6.3830	0.0564	11520.8129	3.0437	0.2570	0.0551
Hungary	5961	3964.0412	3.4650	0.0889	4036.5004	4.4656	0.0535	0.2645
Iceland	2105	1782.3982	4.3145	0.1763	478.7850	7.1262	0.0717	0.1771
India	3621245	6765888.4642	7.1224	0.0410	NA	NA	NA	0.0045
Indonesia	172053	245161.5902	4.7788	0.0316	NA	NA	NA	0.0201
Iran	373570	94307.3179	4.5510	0.1102	279269.4435	2.9052	0.0432	0.0531
Iraq	231177	286347.3795	7.2230	0.0466	NA	NA	NA	0.0971
Ireland	28760	25830.5125	4.2620	0.1063	NA	NA	NA	0.1508
Honduras	60174	16392.4036	4.3321	0.1391	81068.1196	6.1544	0.0895	0.0666
Italy	268366	234107.3748	4.0454	0.0874	152109.5321	4.8147	0.0551	0.1475
Jamaica	2357	NA	NA	NA	NA	NA	NA	NA
Japan	67865	16549.7588	7.8244	0.1231	58877.8030	6.7353	0.0859	0.0472
Kazakhstan	130673	NA	NA	NA	NA	NA	NA	NA
Kenya	34057	44506.1507	6.7577	0.0519	NA	NA	NA	0.0686
Kosovo	13334	19149.3567	5.9962	0.0424	NA	NA	NA	0.1251
Kuwait	84636	67873.6972	5.5275	0.0521	16566.5209	2.7567	0.1815	0.1562
Kyrgyzstan	43898	43306.2180	14.1798	0.1217	NA	NA	NA	1.0229
Lebanon	16870	NA	NA	NA	NA	NA	NA	NA
Luxembourg	8010	50974.5449	8.4007	0.0547	NA	NA	NA	0.0772
Madagascar	14843	3849.0711	3.0137	0.1560	3829.1249	5.4731	0.1022	0.1072
Malawi	5536	15669.2011	9.1087	0.0789	NA	NA	NA	0.0803
Malaysia	9334	427.7014	7.2719	0.2915	5118.5363	3.6000	0.0826	0.0599
Maldives	7667	6372.3634	3.9217	0.1149	2242.5502	2.1243	0.0925	0.2918
Luxembourg	8010	2220.3578	3.0390	0.0913	6468.6466	4.3292	0.0850	0.1165
Mauritania	7022	6640.6373	3.9054	0.0898	NA	NA	NA	0.1372
Mexico	641442	718779.9196	5.0133	0.0393	NA	NA	NA	0.0171
Moldova	36700	30054.5755	3.7987	0.0377	21681.3023	2.9687	0.0787	0.0713
Montenegro	4790	5040.4658	9.0828	0.0713	555.1335	3.0227	0.4366	0.3103
Morocco	61399	NA	NA	NA	NA	NA	NA	NA
Mozambique	3821	8769.4296	4.7192	0.0336	NA	NA	NA	0.0722
Namibia	7365	19752.6493	4.6521	0.0557	NA	NA	NA	0.0369
Nepal	38561	18780.2065	6.6411	0.1098	30689.6636	4.1320	0.1019	0.0727
Netherlands	70071	47727.8050	3.8502	0.0920	21992.4891	4.7197	0.1103	0.0793
New Zealand	1387	1117.4873	3.3022	0.2346	NA	NA	NA	0.0959
Nicaragua	4494	4483.4442	2.0435	0.0494	NA	NA	NA	0.2250
Niger	1176	56771.2201	4.7597	0.0449	NA	NA	NA	0.0283
Nigeria	53865	1555.4972	3.4741	0.1320	12680.0369	3.8436	0.0549	0.0639
Norway	10543	8479.5156	3.2578	0.1023	1761.7727	4.5090	0.1300	0.1522
Oman	85544	89246.7641	6.5958	0.0616	NA	NA	NA	0.0878
Pakistan	295849	294439.8470	6.1567	0.0648	NA	NA	NA	0.0493
Palestine	29063	30248.6952	9.1084	0.0669	NA	NA	NA	0.1613
Panama	92065	117314.2780	4.9029	0.0364	NA	NA	NA	0.0192
Paraguay	17195	NA	NA	NA	NA	NA	NA	NA

Peru	647166	314970.4317	5.4482	0.0677	452678.9916	3.2689	0.0727	0.0345
Philippines	217396	7709617.3043	8.6352	0.0295	NA	NA	NA	0.1425
Poland	66870	37617.3841	3.2568	0.0471	37253.0201	3.3748	0.0810	0.0678
Portugal	57929	27725.1980	3.9706	0.1185	24053.6845	2.7848	0.0663	0.0624
Puerto Rico	32848	13037.7613	3.5594	0.0398	30810.2729	3.9246	0.0771	0.0734
Qatar	118575	114154.2700	5.3173	0.0617	NA	NA	NA	0.0291
Romania	86785	18736.0502	3.8730	0.0867	88299.9668	4.3176	0.0575	0.0456
Russia	990326	944933.9504	4.0525	0.0441	NA	NA	NA	0.1218
Rwanda	4020	8246.7708	4.9188	0.0283	NA	NA	NA	0.7079
Saudi Arabia	314821	320470.6504	5.0217	0.0474	NA	NA	NA	0.0383
Senegal	13556	14520.3585	3.8986	0.0356	NA	NA	NA	0.0869
Serbia	31365	10889.8012	4.6659	0.1250	20573.9984	4.3379	0.0792	0.0281
Singapore	56771	43064.6037	6.9493	0.0763	12182.6864	3.2260	0.1319	0.1479
Slovakia	3876	1502.3267	3.6374	0.1185	5628.8348	4.9385	0.0493	0.1520
South Africa	625056	657770.4040	8.8676	0.0697	NA	NA	NA	0.0165
South Korea	19947	10142.9519	3.1675	0.1737	NA	NA	NA	0.3329
South Sudan	2519	2034.9156	3.9351	0.1254	357.7103	3.2445	0.1936	0.1939
Spain	463943	232080.0683	4.5993	0.1206	519213.3489	5.8299	0.0646	0.1405
Sudan	13189	11334.2564	3.7113	0.0706	1447.2698	3.2426	0.2058	0.0992
Suriname	4009	6441.6655	4.3439	0.0529	NA	NA	NA	0.0399
Sweden	84233	88955.5267	3.6997	0.0388	9247.8436	2.9268	0.1461	0.0963
Switzerland	41906	30089.0565	4.3551	0.1421	16542.9808	4.3765	0.0534	0.0856
Syria	2703	NA	NA	NA	NA	NA	NA	NA
Tajikistan	8550	7809.6500	2.2830	0.0596	NA	NA	NA	0.2877
Thailand	3412	3129.1627	4.0437	0.1363	NA	NA	NA	0.4098
Trinidad and Tobago	1683	NA	NA	NA	NA	NA	NA	NA
Tunisia	3685	994.4882	2.9592	0.1432	NA	NA	NA	0.0916
Turkey	268546	198451.0508	2.9024	0.0638	50313.2130	2.8069	0.1128	0.2541
Uganda	3044	1077.7651	5.6711	0.0898	NA	NA	NA	0.2300
Ukraine	119074	202167.8426	3.9370	0.0252	NA	NA	NA	0.1084
United Arab Emirates	69690	60073.5725	4.2690	0.0514	8839.0863	3.0765	0.1818	0.0853
United Kingdom	334467	277495.8516	4.2224	0.0742	56759.8300	2.9675	0.0747	0.0735
United States of America	5997163	1683559.9636	5.2804	0.0846	4524204.7065	3.5090	0.0614	0.0656
Uzbekistan	41651	71893.9253	5.8737	0.0390	NA	NA	NA	0.0910
Venezuela	45868	101429.9411	7.4504	0.0446	NA	NA	NA	0.1045
Zambia	12025	25961.2086	6.1123	0.0441	NA	NA	NA	0.2871
Zimbabwe	6412	7030.4941	6.1792	0.0805	NA	NA	NA	0.1369

Table S3. Parameter values estimated from the segmented Gompertz model. α is the maximum number of predicted cumulative confirmed cases, β is the time when we start to see a rise in the number of confirmed cases, γ is the increase rate of number of confirmed cases. NA indicates no values returned due to failure of the model to converge.

Segmented Gompertz Model								
		1st Segment			2nd Segment			
**Country**	**Maximum Cumulative cases**	α	β	γ	α	β	γ	**MSSE**
Afghanistan	38162	38658.2222	25.0726	0.0471	NA	NA	NA	0.0609
Albania	9380	NA	NA	NA	NA	NA	NA	NA
Algeria	44146	18438.6969	5.2016	0.0253	44055.5472	5.8284	0.0364	0.0254
Angola	2624	8050.2783	8.3479	0.0185	NA	NA	NA	0.0974
Argentina	401226	4066522.7272	12.0418	0.0096	NA	NA	NA	0.0173
Armenia	43750	49098.8425	16.6368	0.0300	NA	NA	NA	0.0340
Aruba	1997	NA	NA	NA	NA	NA	NA	NA
Australia	25670	6951.4440	25.7079	0.1324	23230.6065	15.4190	0.0505	0.0557
Austria	27218	16155.0090	11.0045	0.1050	52770.0104	5.5194	0.0142	0.0947
Azerbaijan	36309	57315.1635	11.6912	0.0219	3083.2804	4.2461	0.1430	0.1164
Bahamas	2167	5701.9544	75.2460	0.0308	NA	NA	NA	0.1588
Bahrain	51574	66098.9116	11.4035	0.0207	NA	NA	NA	0.0264
Bangladesh	310822	382000.1473	9.6930	0.0240	NA	NA	NA	0.0181
Belarus	71687	71550.0996	10.0480	0.0389	5377.0525	4.1283	0.0834	0.0226
Belgium	85442	60644.2641	9.9910	0.0647	36686.9561	7.1235	0.0421	0.0271
Bolivia	115968	227156.0219	11.1086	0.0177	NA	NA	NA	0.0234
Bosnia and Herze-govina	19789	NA	NA	NA	NA	NA	NA	NA
Brazil	3862311	7198325.5242	10.3171	0.0160	NA	NA	NA	0.0094
Bulgaria	16190	4232.8425	4.5593	0.0290	17992.4458	6.4959	0.0330	0.0421
Cameroon	19142	108707.0966	6.8335	0.0119	6516.6774	3.3445	0.0703	0.2447
Canada	127940	109946.6392	8.5150	0.0420	27876.8064	3.6582	0.0460	0.0235
Cape Verde	3852	833.7525	4.0029	0.0399	5158.9832	4.8344	0.0235	0.0685
Central African Re-public	4700	4833.5213	12.9590	0.0555	NA	NA	NA	0.0936
Chile	409974	415001.2049	29.4866	0.0376	NA	NA	NA	0.1943
China	89868	84708.4919	21.1560	0.1299	NA	NA	NA	0.5822
Colombia	607938	7084799.4030	11.9798	0.0094	NA	NA	NA	0.0533
Congo	3979	6520.0095	8.7962	0.0208	NA	NA	NA	0.3839
Costa Rica	39699	136895.5602	22.2936	0.0170	NA	NA	NA	0.0329
Cote d′ Ivoire	17948	26535.0293	10.5070	0.0226	1163.2906	4.0580	0.1612	0.2006
Croatia	10123	2224.6337	5.2293	0.0869	35557.9695	6.2427	0.0144	0.2303
Cuba	3973	2044.3069	5.4702	0.0707	464.3771	4.3821	0.1089	0.0218
Czechia	24367	8766.2736	6.1152	0.0727	47008.7555	5.0086	0.0155	0.0728
Democratic Republic of the Congo	10044	10736.4218	11.8301	0.0324	274.5472	4.6493	0.2899	0.0508
Denmark	16700	12688.0788	4.8168	0.0481	8468.0207	6.0552	0.0374	0.0926
Djibouti	5385	1128.0306	13.5460	0.1842	4055.6841	8.8338	0.0856	0.2978
Dominican Republic	94241	494048.5687	6.4591	0.0086	NA	NA	NA	0.1698
Ecuador	117639	48988.1455	13.5407	0.0597	118443.9190	4.0637	0.0228	0.2421
Egypt	98727	112100.7245	27.5626	0.0341	3048.1640	3.8200	0.1437	0.0843
El Salvador	25729	84767.4806	8.6124	0.0134	NA	NA	NA	0.1306
Equatorial Guinea	4941	2947.6606	4.7078	0.0329	2242.6633	11.3615	0.1124	0.6882
Estonia	2373	1898.7892	4.0019	0.0674	2204.7769	6.8346	0.0199	0.1693

Eswatini	4561	19034.0804	7.6768	0.0133	NA	NA	NA	0.1214
Ethiopia	51122	NA	NA	NA	NA	NA	NA	NA
Finland	8077	7383.6402	5.3518	0.0485	2396.7520	5.6497	0.0314	0.0416
France	278709	151791.8661	12.8923	0.0683	NA	NA	NA	0.0523
Gabon	8505	6670.3765	8.3496	0.0416	3268.2183	4.3985	0.1016	0.0552
Gambia	2963	4460.8609	16.3888	0.0590	NA	NA	NA	0.0822
Georgia	1487	180590.9806	14.3623	0.0745	1399965.8160	6.0875	0.0076	0.0475
Germany	242381	43428.0235	8.2229	0.0245	50878.8766	5.1497	0.0303	0.1860
Greece	10134	2850.2120	5.6666	0.0705	NA	NA	NA	0.0592
Guam	1347	NA	NA	NA	NA	NA	NA	NA
Guatemala	73912	120849.4230	12.8995	0.0215	NA	NA	NA	0.0557
Guinea	9371	6276.1720	4.8603	0.0356	5896.6630	3.6876	0.0353	0.0422
Haiti	8209	7919.9844	14.2064	0.0522	NA	NA	NA	0.0596
Honduras	60174	94977.9591	13.7684	0.0214	16635.0074	4.0325	0.1127	0.0782
Hungary	5961	4158.2722	6.0539	0.0554	90891.8764	7.8593	0.0089	0.2417
Iceland	2105	1815.9673	11.4017	0.1168	6308.7422	11.2834	0.0124	0.2764
India	3621245	87187036.6787	11.8631	0.0075	NA	NA	NA	0.0204
Indonesia	172053	798318.0620	7.0186	0.0087	NA	NA	NA	0.0084
Iran	373570	118567.0986	7.9497	0.0523	348087.8281	3.8623	0.0216	0.0349
Iraq	231177	565000.6425	17.3186	0.0163	NA	NA	NA	0.0389
Ireland	28760	26103.4687	11.1085	0.0707	NA	NA	NA	0.1597
Honduras	60174	17138.7762	10.4599	0.0872	125660.9635	13.5198	0.0359	0.0430
Italy	268366	239477.4394	9.4656	0.0574	NA	NA	NA	0.0219
Jamaica	2357	NA	NA	NA	NA	NA	NA	NA
Japan	67865	17531.5024	89.4251	0.0760	98622.3568	15.6933	0.0325	0.0955
Kazakhstan	130673	NA	NA	NA	NA	NA	NA	NA
Kenya	34057	99124.7710	13.0280	0.0165	NA	NA	NA	0.1973
Kosovo	13334	79700.2167	9.2221	0.0103	NA	NA	NA	0.2460
Kuwait	84636	82441.1292	14.3643	0.0264	20197.2297	3.6386	0.0935	0.0725
Kyrgyzstan	43898	45547.7268	5355.3572	0.0766	NA	NA	NA	1.2727
Lebanon	16870	NA	NA	NA	NA	NA	NA	NA
Luxem-bourg	8010	NA	NA	NA	NA	NA	NA	NA
Madagascar	14843	3911.1573	5.0182	0.1050	4326.7213	15.6462	0.0556	0.0534
Malawi	5536	18617.7675	91.9470	0.0405	NA	NA	NA	0.2242
Malaysia	9334	560.8286	29.4977	0.1370	5847.5530	5.6631	0.0448	0.0626
Maldives	7667	6865.9962	7.4158	0.0678	2302.4141	2.8881	0.0620	0.2145
Luxem-bourg	8010	2481.8562	4.3299	0.0516	13064.4175	6.1831	0.0290	0.0949
Mauritania	7022	6952.5768	7.9523	0.0559	NA	NA	NA	0.1181
Mexico	641442	1078940.7137	8.7075	0.0161	NA	NA	NA	0.0054
Moldova	36700	89476.8616	5.3770	0.0109	33783.2559	3.8986	0.0326	0.0632
Montenegro	4790	7276.4251	58.9567	0.0315	764.5359	4.0296	0.1998	0.3762
Morocco	61399	NA	NA	NA	NA	NA	NA	NA
Mozam-bique	3821	87202.3592	7.5356	0.0066	NA	NA	NA	0.0683
Namibia	7365	333497.1922	7.8533	0.0098	NA	NA	NA	0.0363
Nepal	38561	23759.6419	21.5334	0.0521	114201.0768	5.9905	0.0269	0.1086
Netherlands	70071	49107.7894	7.9913	0.0590	38774.5904	7.2102	0.0405	0.0432
New Zea-land	1387	1127.1035	6.1202	0.1598	NA	NA	NA	0.0097
Nicaragua	4494	4915.0570	2.6343	0.0300	NA	NA	NA	0.1603
Niger	1176	73615.8370	8.6526	0.0208	NA	NA	NA	0.0222
Nigeria	53865	1874.5592	4.9774	0.0664	15364.7097	6.1098	0.0278	0.0358
Norway	10543	8618.0392	5.7781	0.0680	2843.9925	6.8781	0.0507	0.0812
Oman	85544	100763.2996	28.3321	0.0334	NA	NA	NA	0.1751

Pakistan	295849	313983.3281	28.4530	0.0386	NA	NA	NA	0.1366
Palestine	29063	42050.0663	59.6946	0.0299	NA	NA	NA	0.0633
Panama	92065	260971.5242	7.2872	0.0116	NA	NA	NA	0.0598
Paraguay	17195	NA	NA	NA	NA	NA	NA	NA
Peru	647166	446214.4655	10.7987	0.0290	846683.9627	4.3642	0.0264	0.0321
Philippines	217396	NA	NA	NA	NA	NA	NA	NA
Poland	66870	47514.4286	4.5190	0.0231	64104.7074	4.5054	0.0308	0.0361
Portugal	57929	29936.8951	7.6538	0.0699	29015.3918	3.6595	0.0343	0.0215
Puerto Rico	32848	65040.0602	5.4168	0.0096	50324.6920	5.5172	0.0301	0.0745
Qatar	118575	120095.6164	18.2530	0.0378	NA	NA	NA	0.0333
Romania	86785	21563.1947	6.5107	0.0466	216103.4983	6.0951	0.0177	0.0318
Russia	990326	1057244.6747	7.5490	0.0247	NA	NA	NA	0.0380
Rwanda	4020	160397.3892	8.3458	0.0048	NA	NA	NA	0.6512
Saudi Arabia	314821	375527.3377	11.0682	0.0244	NA	NA	NA	0.0589
Senegal	13556	19817.4519	5.8206	0.0161	NA	NA	NA	0.0395
Serbia	31365	11544.5233	11.9676	0.0755	24299.8019	7.8757	0.0408	0.0301
Singapore	56771	46555.2356	44.9176	0.0451	13163.0862	4.8592	0.0772	0.0811
Slovakia	3876	1564.7214	6.4994	0.0733	74539.1570	8.0670	0.0091	0.1994
South Africa	625056	787210.6110	75.3165	0.0351	NA	NA	NA	0.1231
South Korea	19947	10364.8855	5.4689	0.1147	NA	NA	NA	0.1889
South Sudan	2519	2147.1872	7.8644	0.0767	373.3645	5.1459	0.1199	0.2220
Spain	463943	237938.9846	13.3300	0.0784	NA	NA	NA	0.0279
Sudan	13189	12339.0733	6.4342	0.0410	1902.2561	4.4326	0.0969	0.0612
Suriname	4009	29831.0627	6.3839	0.0128	NA	NA	NA	0.0792
Sweden	84233	124252.7248	5.2176	0.0170	11067.5839	3.9308	0.0759	0.1109
Switzerland	41906	30687.3318	11.6364	0.0936	56221.8662	6.3637	0.0146	0.0491
Syria	2703	NA	NA	NA	NA	NA	NA	NA
Tajikistan	8550	8127.3560	3.2065	0.0399	NA	NA	NA	0.1665
Thailand	3412	3154.1186	9.3739	0.0897	NA	NA	NA	0.2209
Trinidad and Tobago	1683	NA	NA	NA	NA	NA	NA	NA
Tunisia	3685	1038.2063	4.5857	0.0915	NA	NA	NA	0.0306
Turkey	268546	207933.5885	4.5240	0.0409	68974.9480	3.6475	0.0517	0.1452
Uganda	3044	1188.0377	19.7095	0.0514	NA	NA	NA	0.1800
Ukraine	119074	NA	NA	NA	NA	NA	NA	NA
United Arab Emirates	69690	67678.3664	8.2582	0.0283	13578.4568	4.0506	0.0756	0.0398
United Kingdom	334467	291405.6852	9.4917	0.0458	99004.9490	3.9148	0.0286	0.0251
United States of America	5997163	1987478.8437	13.0915	0.0439	5952114.1576	4.8924	0.0284	0.0231
Uzbekistan	41651	788275.6765	9.1565	0.0072	NA	NA	NA	0.2148
Venezuela	45868	3551978.5884	12.7690	0.0067	NA	NA	NA	0.1627
Zambia	12025	31660320.7299	13.1480	0.0038	NA	NA	NA	0.4628
Zimbabwe	6412	10189.0969	14.4420	0.0338	NA	NA	NA	0.1983

Supplementary Information Text

Correlation analysis between the log-scaled parameters of the Growth curve models.

Pearson correlation analysis for segmented Logistic and Gompertz models with the log-scaled of parameters are performed to determine the similarity between the parameters across the two models. Before performing the linear regression for the growth curve

model parameters and the national indicator variables, we interpret the correlation coefficients between model parameters and across segments. The points of focus are whether the parameter differences is retained in both Logistic and Gompertz models, and whether the parameters α, β and γ are correlated across segments.

For Logistic models (**Figure S**3), correlation analysis between parameters α_1 and α_2, the maximum number of predicted cumulative confirmed cases in the 1st segment and 2nd segment respectively, have a strong positive correlation of 0.7. On the other hand, it was found that there was little correlation between the parameters β_1 and β_2 (-0.089) and in γ_1 and γ_2(-0.32). While for Gompertz models (**Figure S4**), similar with Logistic model, two parameters α_1 and α_2, the maximum number of predicted cumulative confirmed cases in the 1st segment and 2nd segment respectively, have a strong positive correlation (0.57) while there was no correlation between the coefficients β_1 and β_2(0.15), also in γ_1 and γ_2(-0.34).

The parameters β_2 and γ_2 have little correlation (-0.069 and 0.077 in the 1st segment, -0.27 and -0.31 in the 2nd segment for Logistic and Gompertz models) that is negligible when analyzing correlation coefficients. However, in the 1st segment, the correlation coefficients between parameters α_1 and γ_1 for both Logistic (-0.52) and Gompertz (-0.66) models show that the relationship between the cumulative confirmed cases and the slope of the model has a negative correlation. It is same with the parameters α_2 and γ_2 (-0.55 for Logistic model and -0.7 for Gompertz model) in the 2nd segment (**Figure S5** and **S6**).

We can interpret the negative correlation between α and γ with two aspects. The reason for this correlation is because of the internal structure of GCMs. Also, maybe social distancing policies that the countries implemented contributed to observed negative correlation. In other words, when COVID-19 had out broken at first in February and March in 2020, the policies was valid to reduce daily cases. The second one is the opposite case of the above reason. In the countries having had slight incline for COVID-19, they dealt with it relatively easily, so the level of social distancing policies was lower. It caused bad results that the cumulative cases had been continuously increasing. Therefore, this analysis confirms that the parameters have similar interpretation across models.

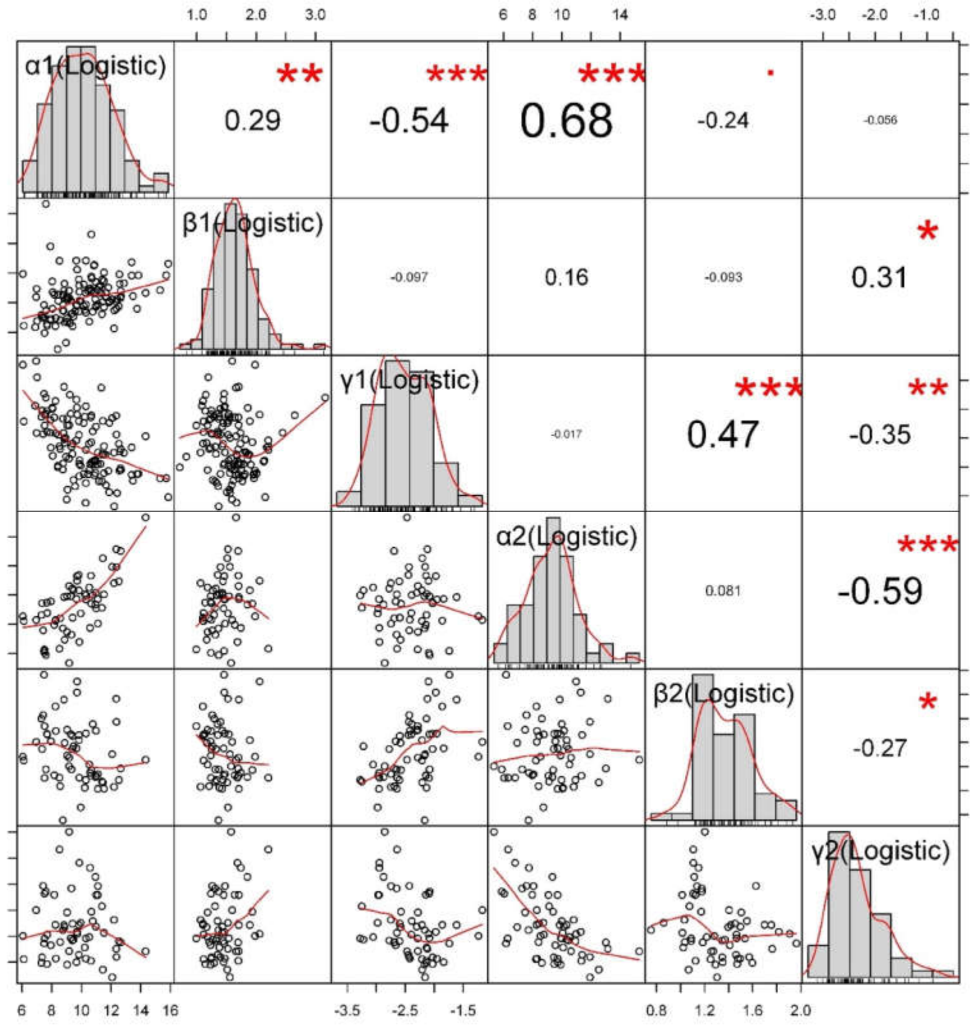

Figure S3. Comparison of Logistic Model (log-scaled) parameters $\boldsymbol{\alpha}$, $\boldsymbol{\beta}$, and $\boldsymbol{\gamma}$ in the 1st and 2nd segments.

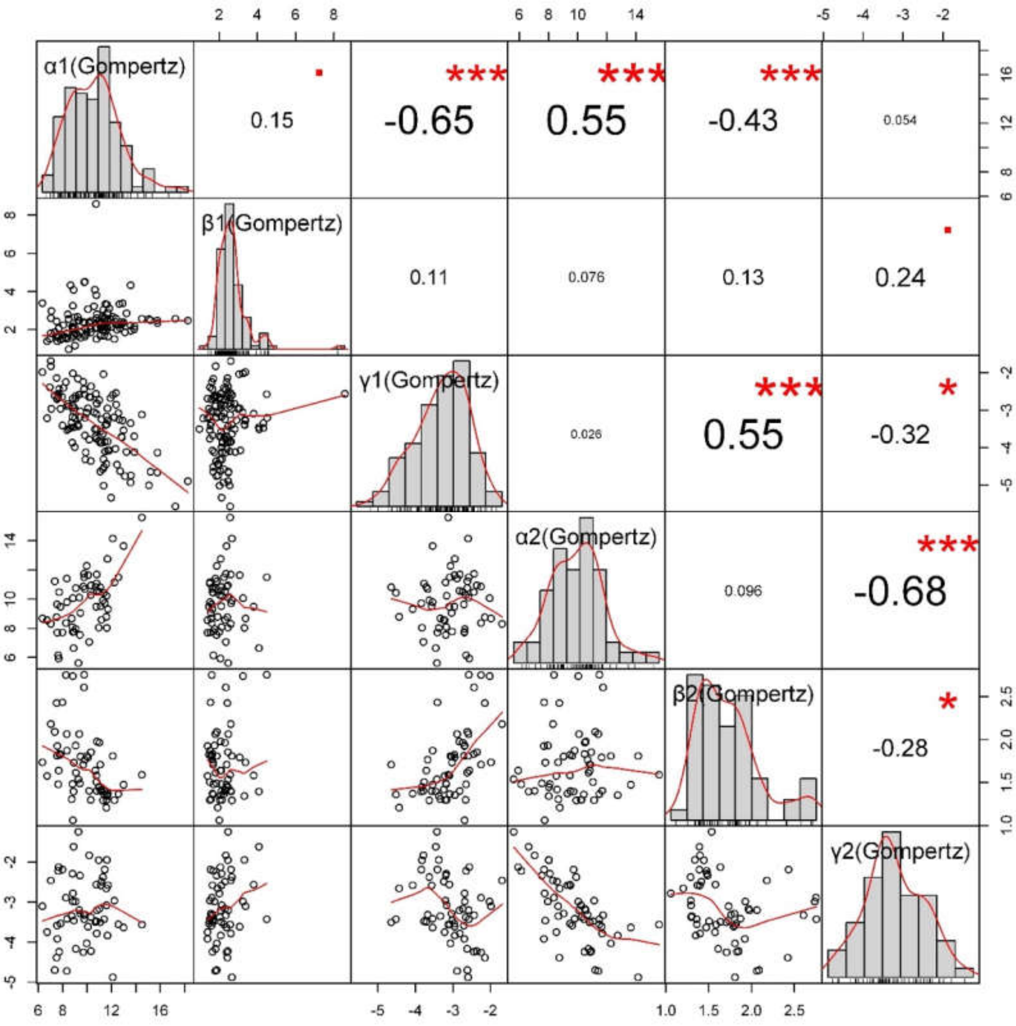

Figure S4. Comparison of Gompertz Model (log-scaled) parameters $\boldsymbol{\alpha}$, $\boldsymbol{\beta}$, and $\boldsymbol{\gamma}$ in the 1st and 2nd segments.

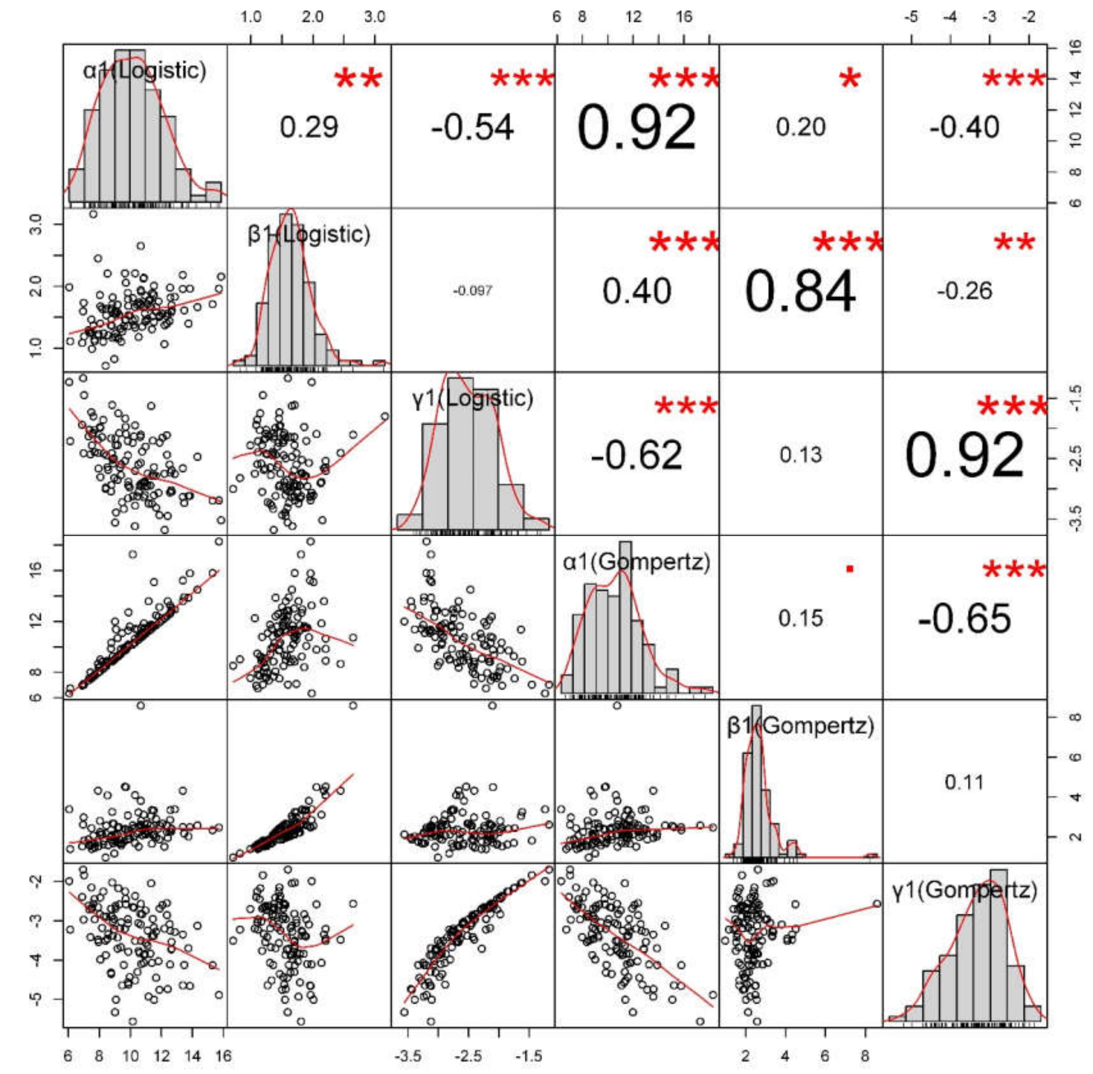

Figure S5. Comparison of Logistic and Gompertz Model (log-scaled) parameters $\boldsymbol{\alpha}$, $\boldsymbol{\beta}$, and $\boldsymbol{\gamma}$ in the 1st segment.

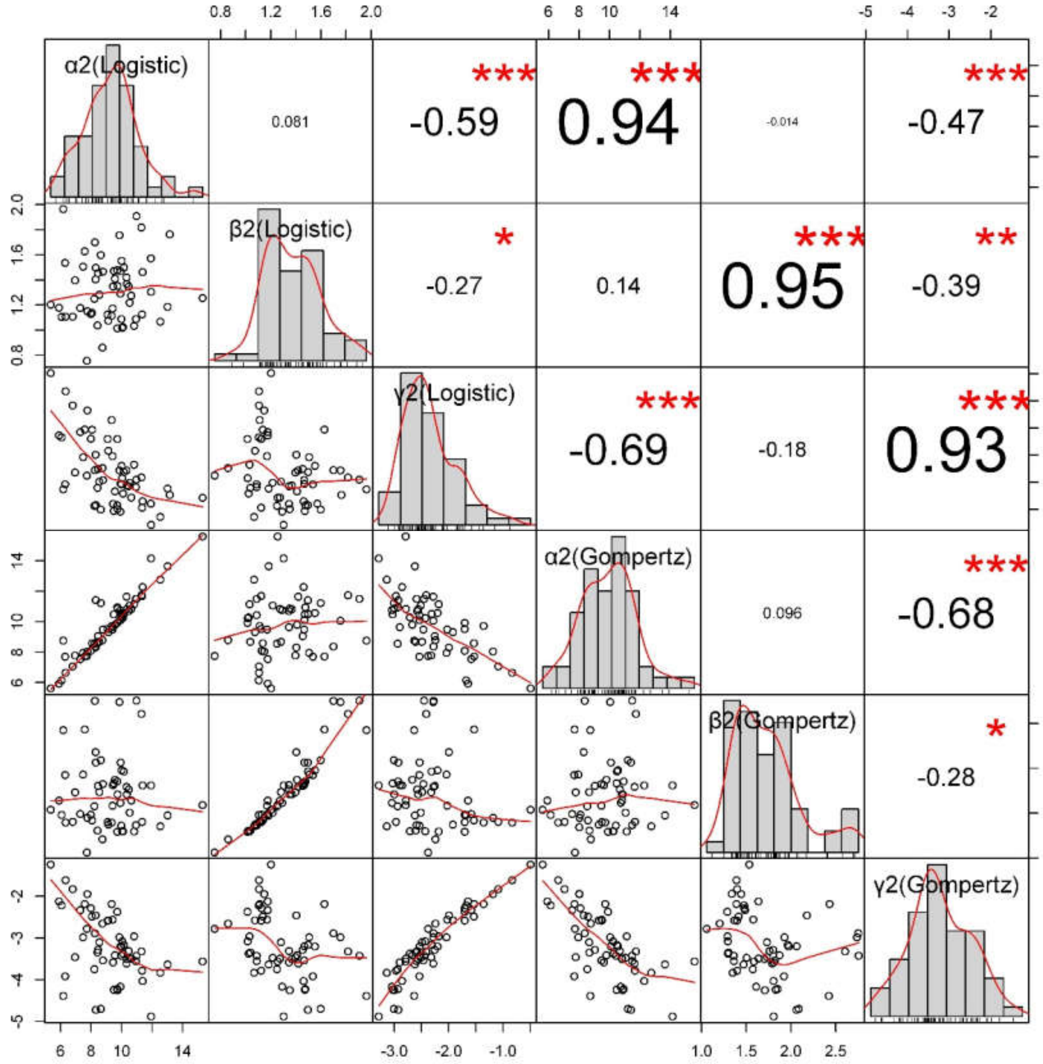

Figure S6. Comparison of Logistic and Gompertz Model (log-scaled) parameters $\boldsymbol{\alpha}$, $\boldsymbol{\beta}$, and $\boldsymbol{\gamma}$ in the 2nd segment.

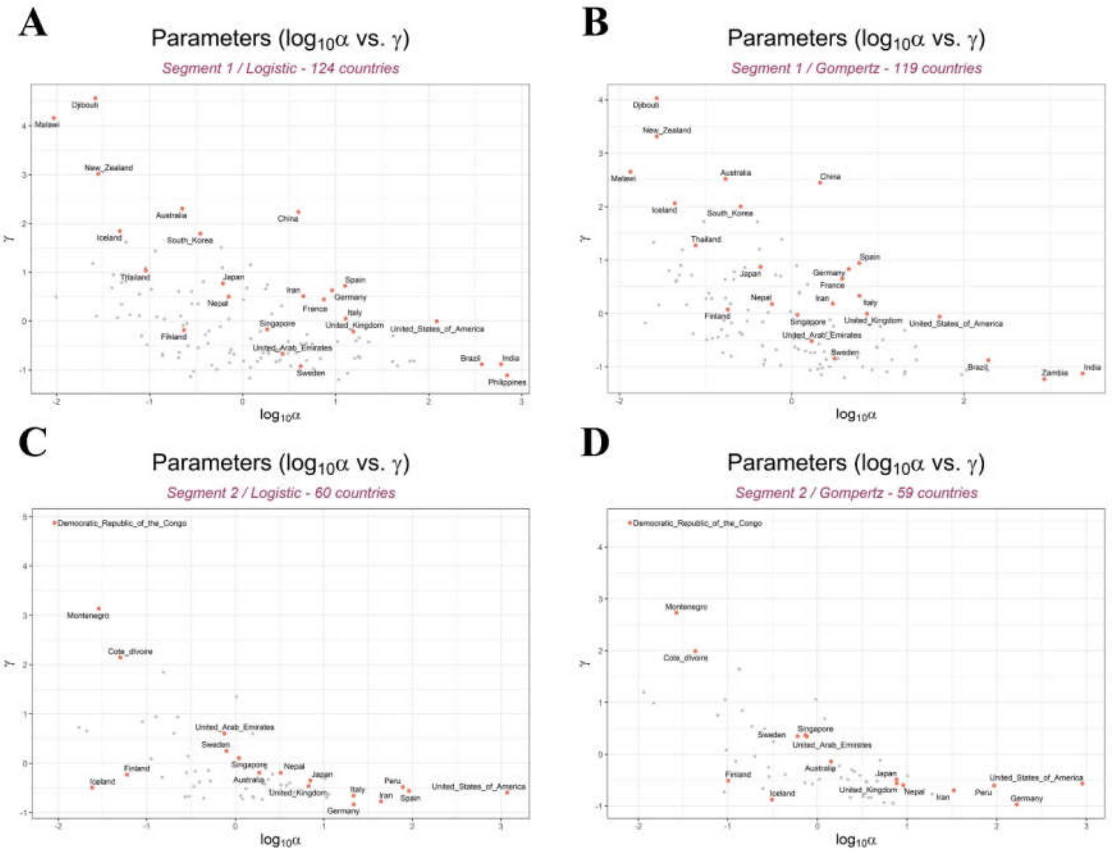

Figure S7. Variation of log10 maximum predicted cumulative cases (**α**) and rate of spread of COVID-19 among countries (**γ**).

Table S4. A. Results from the regression analysis of parameters of growth curve models and national factors for the 1st segment. The p-values are in the brackets.

Results of Linear regression of parameters of growth curve models and national factors				
Sample size	Factor\Parameter		1st Segment Logistic	1st Segment Gompertz
113	Child Vaccination rate	α	-162866.78 (0.1007)	-87309.168 (0.9204)
		β	-0.2525 (0.099)	-1.7217 (0.2962)
		γ	0.0092 (0.0474)	0.0063 (0.0646)
81	Malnutrition rate	α	148742.297 (0.2794)	1106109.04 (0.3232)
		β	0.6544 (8.18E-05)	4.7293 (0.0103)
		γ	-0.0135 (0.0046)	-0.01125 (0.0013)
114	Migration rate	α	-24299.073 (0.8057)	-198330.74 (0.8031)
		β	0.016 (0.9168)	0.9332 (0.5355)
		γ	0.0055 (0.2363)	0.0054 (0.0806)

110	Proportion of urban population	α	-76419.969<br>(0.4555)	-1091047.7<br>(0.1873)
		β	-0.1491<br>(0.331)	0.0504<br>(0.9734)
		γ	0.0043<br>(0.3689)	0.0048<br>(0.1402)
21	Public Social Welfare (2018)	α	30467.2057<br>(0.062)	32787.2884<br>(0.0529)
		β	0.02383<br>(0.8547)	0.0074<br>(0.9919)
		γ	-0.0133<br>(0.1745)	-0.0092<br>(0.1876)
46	National Competitive-ness (2020)	α	430277.632<br>(0.0667)	1872125.73<br>(0.3459)
		β	0.03348<br>(0.1389)	0.589<br>(0.8188)
		γ	-0.0162<br>(0.0184)	-0.0126<br>(0.0148)
89	Temperature	α	197487.26<br>(0.1151)	699067.536<br>(0.509)
		β	0.3798<br>(0.0165)	1.537<br>(0.3739)
		γ	-0.0069<br>(0.2258)	-0.0076<br>(0.051)
89	Rain	α	271748.495<br>(0.0284)	-20108.621<br>(0.9856)
		β	0.339<br>(0.0321)	2.1164<br>(0.2413)
		γ	-0.003<br>(0.6056)	-0.003<br>(0.465)
24	Pharmacy Sales (2018)	α	27315.7182<br>(0.2034)	27408.8334<br>(0.2288)
		β	0.7456<br>(0.0004)	12.2806<br>(0.0001)
		γ	-0.0033<br>(0.7381)	-0.0028<br>(0.6785)
89	Exportation (2016)	α	-185522.7<br>(0.1413)	-1013900.6<br>(0.322)
		β	-0.337587<br>(0.0467)	-0.9287<br>(0.5576)
		γ	0.0064<br>(0.1579)	0.0068<br>(0.0377)
89	Importation (2016)	α	-93249.215<br>(0.4609)	-1272023.9<br>(0.219)
		β	-0.4066<br>(0.0158)	-2.9847<br>(0.0602)
		γ	-0.0013<br>(0.7728)	0.0016<br>(0.626)
113	Total GDP (2018)	α	-21693.095<br>(0.8283)	-88251.311<br>(0.9139)
		β	0.0277<br>(0.8578)	-0.501<br>(0.7455)
		γ	-0.0018<br>(0.708)	0.0007<br>(0.8238)
46	Travel In (2017)	α	188185.298<br>(0.4312)	-1212922.4<br>(0.5466)
		β	0.1928	-0.367

			(0.3844)	(0.8885)
		γ	0.0058<br>(0.3608)	0.003<br>(0.497)
46	Travel Out (2017)	α	166194.703<br>(0.4904)	986646.17<br>(0.6203)
		β	-0.0916<br>(0.6826)	-0.6687<br>(0.7962)
		γ	-0.0126<br>(0.0422)	-0.0088<br>(0.04)
114	Aging Index (2020)	α	-87431.181<br>(0.3755)	-529763.62<br>(0.5089)
		β	-0.5072<br>(0.0007)	0.0891<br>(0.9533)
		γ	0.0119<br>(0.0094)	0.0121<br>6.83E-05)
112	GDP per Capita (2019)	α	-3.8816<br>(0.3797)	-28.9139<br>(0.4185)
		β	-1.49E-05<br>(0.0278)	4.27E-06<br>(0.9497)
		γ	6.36E-07<br>(0.0018)	5.86E-07<br>(1.38E-05)
114	Birth Rate (2020)	α	-71328.528<br>(0.471)	-301881.37<br>(0.7092)
		β	0.2595<br>(0.0886)	0.323<br>(0.8332)
		γ	-0.0059<br>(0.2088)	-0.0072<br>(0.0235)
112	Population Density	α	16491.3143<br>(0.9336)	324103.636<br>(0.8381)
		β	0.3404<br>(0.2862)	5.7412<br>(0.0636)
		γ	-0.0026<br>(0.7878)	-0.0006<br>(0.9235)
114	Population	α	144085.33<br>(5.13E-08)	30147994.4<br>(4.07E-48)
		β	0.565<br>(0.2136)	0.8402<br>(0.8906)
		γ	0.0053<br>(0.6978)	-0.0218<br>(0.082)
118	Median Age	α	-40858.707<br>(0.6693)	-293347.94<br>(0.7069)
		β	-0.5119<br>(0.0007)	-1.0658<br>(0.4869)
		γ	0.0103<br>(0.0233)	0.0112<br>(0.0002)
117	Aged 65 older	α	-69195.517<br>(0.4445)	-444210.9<br>(0.5464)
		β	-0.5127<br>(0.0003)	-0.6779347<br>(0.6398)
		γ	0.01183<br>(0.0058)	0.01193<br>(3.24E-05)
116	Aged 70 older	α	-76963.694<br>(0.395)	-490243.82<br>(0.5058)
		β	-0.5203<br>(0.0003)	-0.7467<br>(0.6064)
		γ	0.0114<br>(0.0076)	0.0118<br>(4.04E-05)

117	GDP per Capita	α	-72600.451<br>(0.4153)	-578839.61<br>(0.4211)
		β	-0.2912<br>(0.0411)	-0.1546<br>(0.913)
		γ	0.0074<br>(0.0823)	0.0083<br>(0.004)
83	Extreme poverty	α	-20797.551<br>(0.8536)	621679.112<br>(0.6259)
		β	0.529<br>(0.0059)	5.5029<br>(0.0038)
		γ	0.0013<br>(0.8401)	-0.0022<br>(0.6292)
117	Cardiovascular Death	α	35364.2112<br>(0.7108)	151720.751<br>(0.8419)
		β	0.1663<br>(0.2642)	0.6245<br>(0.6763)
		γ	-0.0111<br>(0.0111)	-0.0082<br>(0.0066)
117	Diabetes prevalence	α	83615.5532<br>(0.4938)	774400.837<br>(0.4333)
		β	0.2144<br>(0.277)	1.3588<br>(0.4846)
		γ	-0.0106<br>(0.0704)	-0.0065<br>(0.1028)
95	Female Smokers	α	-80878.09<br>(0.4724)	-843124.81<br>(0.3531)
		β	-0.4377<br>(0.0044)	-0.5464<br>(0.7142)
		γ	0.0087<br>(0.0932)	0.0095<br>(0.0057)
93	Male Smokers	α	-65256.249<br>(0.6028)	-1056404.7<br>(0.3089)
		β	-0.2687<br>(0.1202)	-0.5804<br>(0.7338)
		γ	-0.0042<br>(0.468)	-0.0004<br>(0.9236)
52	Handwashing Facilities	α	204992.594<br>(0.325)	467873.18<br>(0.7988)
		β	0.0679<br>(0.7639)	-0.3347<br>(0.887)
		γ	-0.0066<br>(0.2888)	-0.001<br>(0.7875)
105	Hospital Beds per Thousand	α	-144568.79<br>(0.157)	-947703.31<br>(0.2634)
		β	-0.4366<br>(0.0052)	0.1302<br>(0.9351)
		γ	0.0091<br>(0.0592)	0.009<br>(0.0061)
117	Life Expectancy	α	-26753.176<br>(0.7852)	-410006.48<br>(0.6054)
		β	-0.3501<br>(0.0257)	-0.5765<br>(0.7119)
		γ	0.0096<br>(0.0401)	0.0102<br>(0.0012)
116	Human Development Index	α	-11471.282<br>(0.9091)	-452191.27<br>(0.5758)
		β	-0.3341	-0.6506

			(0.0372)	(0.682)
		γ	0.0062<br>(0.1997)	0.008<br>(0.0124)

Table S5. B. Results from the regression analysis of parameters of growth curve models and national factors for the 2nd segment.

Results of Linear regression of parameters of growth curve models and national factors				
**Sample size**	**Factor\Parameter**		**2nd Segment**	
			**Logistic**	**Gompertz**
113	Child Vaccination rate	α	22766.7187<br>(0.8298)	25628.9405<br>(0.8583)
		β	0.3063<br>(0.0962)	0.8224<br>(0.1163)
		γ	-0.0242<br>(0.1748)	-0.0126<br>(0.1667)
81	Malnutrition rate	α	-94847.6759<br>(0.6724)	-140310.632<br>(0.6461)
		β	-0.6739<br>(0.0723)	-1.6175<br>(0.1359)
		γ	0.0398<br>(0.1189)	0.0241<br>(0.0793)
114	Migration rate	α	14085.0612<br>(0.8577)	36207.377<br>(0.7335)
		β	0.0952<br>(0.4904)	0.5389<br>(0.1661)
		γ	-0.0037<br>(0.7824)	-0.0022<br>(0.7476)
110	Proportion of urban population	α	61101.784<br>(0.4972)	80948.5367<br>(0.499)
		β	0.2846<br>(0.0661)	0.9605<br>(0.0251)
		γ	-0.0015<br>(0.875)	-0.0021<br>(0.6895)
21	Public Social Welfare (2018)	α	26122.6436<br>(0.4085)	58126.9043<br>(0.5323)
		β	-0.348<br>(0.1826)	-0.7516<br>(0.3451)
		γ	0.0082<br>(0.2895)	0.0089<br>(0.0866)
46	National Competitive-ness (2020)	α	37310.1995<br>(0.1115)	36555.3449<br>(0.5367)
		β	0.0733<br>(0.7601)	-0.1454<br>(0.8468)
		γ	-0.019<br>(0.0015)	-0.0106<br>(0.0073)
89	Temperature	α	-21204.8904<br>(0.1513)	-36660.1652<br>(0.2723)
		β	-0.2805<br>(0.0689)	-0.5364<br>(0.2332)
		γ	0.0181<br>(0.0471)	0.0092<br>(0.0712)
89	Rain	α	-29052.6804<br>(0.081)	-45051.5504<br>(0.2293)
		β	-0.2067<br>(0.2422)	-0.2352<br>(0.6443)
		γ	0.0145	0.0069

			(0.1653)	(0.2324)
24	Pharmacy Sales (2018)	α	43759.1067<br>(0.0733)	175788.3545<br>(0.0066)
		β	0.2278<br>(0.3962)	0.9545<br>(0.2885)
		γ	-0.006<br>(0.3619)	-0.0016<br>(0.7302)
89	Exportation (2016)	α	-106962.981<br>(0.212)	-123682.78<br>(0.2838)
		β	-0.0232<br>(0.8736)	-0.0896<br>(0.8287)
		γ	-0.0034<br>(0.6923)	-0.0042<br>(0.3716)
89	Importation (2016)	α	-193259.914<br>(0.0496)	-249104.359<br>(0.0621)
		β	-0.1065<br>(0.529)	-0.3803<br>(0.432)
		γ	-0.0037<br>(0.7123)	-0.0063<br>(0.253)
113	Total GDP (2018)	α	-70033.5848<br>(0.3859)	-76160.8968<br>(0.4931)
		β	0.094<br>(0.5079)	0.3571<br>(0.3822)
		γ	0.0212<br>(0.1171)	0.0074<br>(0.2968)
46	Travel In (2017)	α	239740.8481<br>(0.1434)	288980.8722<br>(0.1988)
		β	0.1355<br>(0.5094)	0.4249<br>(0.4799)
		γ	0.0041<br>(0.5463)	0.0115<br>(0.0378)
46	Travel Out (2017)	α	204980.2878<br>(0.1889)	345861.2615<br>(0.0987)
		β	-0.2364<br>(0.2201)	-0.5431<br>(0.3354)
		γ	-0.0004<br>(0.9499)	-0.0029<br>(0.5968)
114	Aging Index (2020)	α	19947.5993<br>(0.7873)	50389.1524<br>(0.6239)
		β	0.4186<br>(0.0007)	0.9256<br>(0.0121)
		γ	-0.0335<br>(0.0058)	-0.0201<br>(0.0014)
112	GDP per Capita (2019)	α	4.5781<br>(0.1479)	6.4778<br>(0.1279)
		β	1.81E-05<br>(0.0006)	5.48E-05<br>(0.0002)
		γ	-9.13E-07<br>(0.0851)	-5.27E-07<br>(0.0482)
114	Birth Rate (2020)	α	-42729.883<br>(0.6268)	-69051.085<br>(0.562)
		β	-0.3307<br>(0.0291)	-0.674<br>(0.1215)
		γ	0.0529<br>(0.0002)	0.0298<br>(2.38E-05)
112	Population Density	α	-35295.5568<br>(0.7692)	-47977.7212<br>(0.7685)

		β	-0.057<br>(0.7877)	-0.0689<br>(0.9088)
		γ	-0.0013<br>(0.9509)	0.0017<br>(0.869)
114	Population	α	5718315.169<br>(9.58E-18)	7460122.241<br>(1.33E-16)
		β	-0.2749<br>(0.8613)	-1.1855<br>(0.7871)
		γ	-0.0211<br>(0.8894)	0.0233<br>(0.7626)
118	Median Age	α	36156.7095<br>(0.6451)	70324.4081<br>(0.5157)
		β	0.3978<br>(0.0029)	0.8141<br>(0.0381)
		γ	-0.0394<br>(0.0022)	-0.0239<br>(0.0003)
117	Aged 65 older	α	35926.7119<br>(0.6034)	69689.3055<br>(0.4656)
		β	0.4287<br>(0.0002)	0.9415<br>(0.0058)
		γ	-0.0324<br>(0.0045)	-0.0199<br>(0.0007)
116	Aged 70 older	α	33132.9659<br>(0.6302)	72072.3096<br>(0.4525)
		β	0.4236<br>(0.0002)	0.0345<br>(0.0063)
		γ	-0.0318<br>(0.0051)	-0.0198<br>(0.0008)
117	GDP per Capita	α	81960.1179<br>(0.2553)	118502.3014<br>(0.2222)
		β	0.2956<br>(0.0171)	0.9188<br>(0.0084)
		γ	-0.0197<br>(0.1047)	-0.0114<br>(0.066)
83	Extreme poverty	α	-76235.7429<br>(0.5732)	-97505.2165<br>(0.5896)
		β	-0.2832<br>(0.1732)	-0.5634<br>(0.3393)
		γ	0.0697<br>(0.0004)	0.0341<br>(0.0007)
117	Cardiovascular Death	α	-76655.7457<br>(0.3483)	-107128.767<br>(0.3143)
		β	-0.3666<br>(0.0091)	-0.9235<br>(0.0164)
		γ	0.0389<br>(00039)	0.0203<br>(0.0022)
117	Diabetes prevalence	α	101926.4412<br>(0.3388)	112135.2213<br>(0.4086)
		β	-0.4435<br>(0.016)	-1.069<br>(0.0298)
		γ	0.0126<br>(0.49)	0.0128<br>(0.143)
95	Female Smokers	α	24766.822<br>(0.7737)	54577.83<br>(0.6384)
		β	0.2858<br>(0.0442)	0.5133<br>(0.2174)
		γ	-0.0025	-0.0056

			(0.7954)	(0.2825)
93	Male Smokers	α	-89380.7136<br>(0.4668)	-109773.08<br>(0.4962)
		β	-0.2111<br>(0.3013)	-0.7053<br>(0.2217)
		γ	0.0319<br>(0.0139)	0.0176<br>(0.0109)
52	Handwashing Facilities	α	6579.7371<br>(0.2045)	7136.3648<br>(0.4027)
		β	0.1928<br>(0.1339)	0.2958<br>(0.2666)
		γ	-0.041<br>(0.2382)	-0.0172<br>(0.2845)
105	Hospital Beds per Thousand	α	-41472.5662<br>(0.6042)	-13167.3972<br>(0.9029)
		β	0.3215<br>(0.0182)	0.9459<br>(0.0141)
		γ	-0.0047<br>(0.6166)	-0.0065<br>(0.2003)
117	Life Expectancy	α	50338.9611<br>(0.5402)	79085.338<br>(0.4844)
		β	0.4937<br>(0.0003)	1.1331<br>(0.005)
		γ	-0.0447<br>(0.0008)	-0.0265<br>(0.0001)
116	Human Development Index	α	85588.3356<br>(0.2845)	131772.9588<br>(0.2257)
		β	0.4132<br>(0.0024)	0.9193<br>(0.0197)
		γ	-0.0401<br>(0.0022)	-0.0238<br>(0.0004)

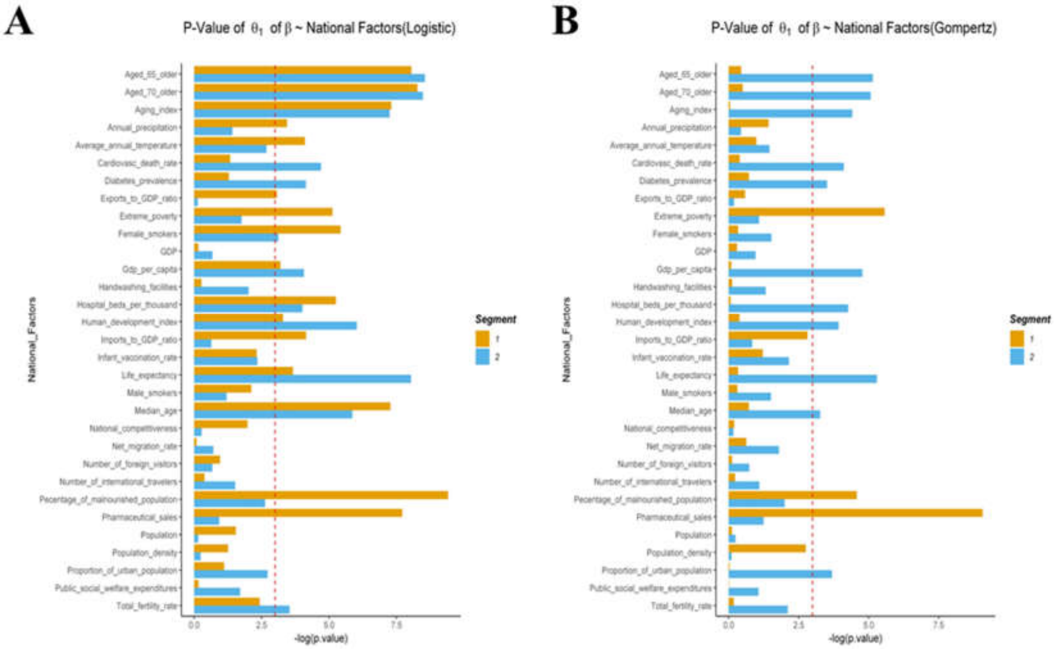

Figure S8. *P*-values of the relationship between national factors and **β**.

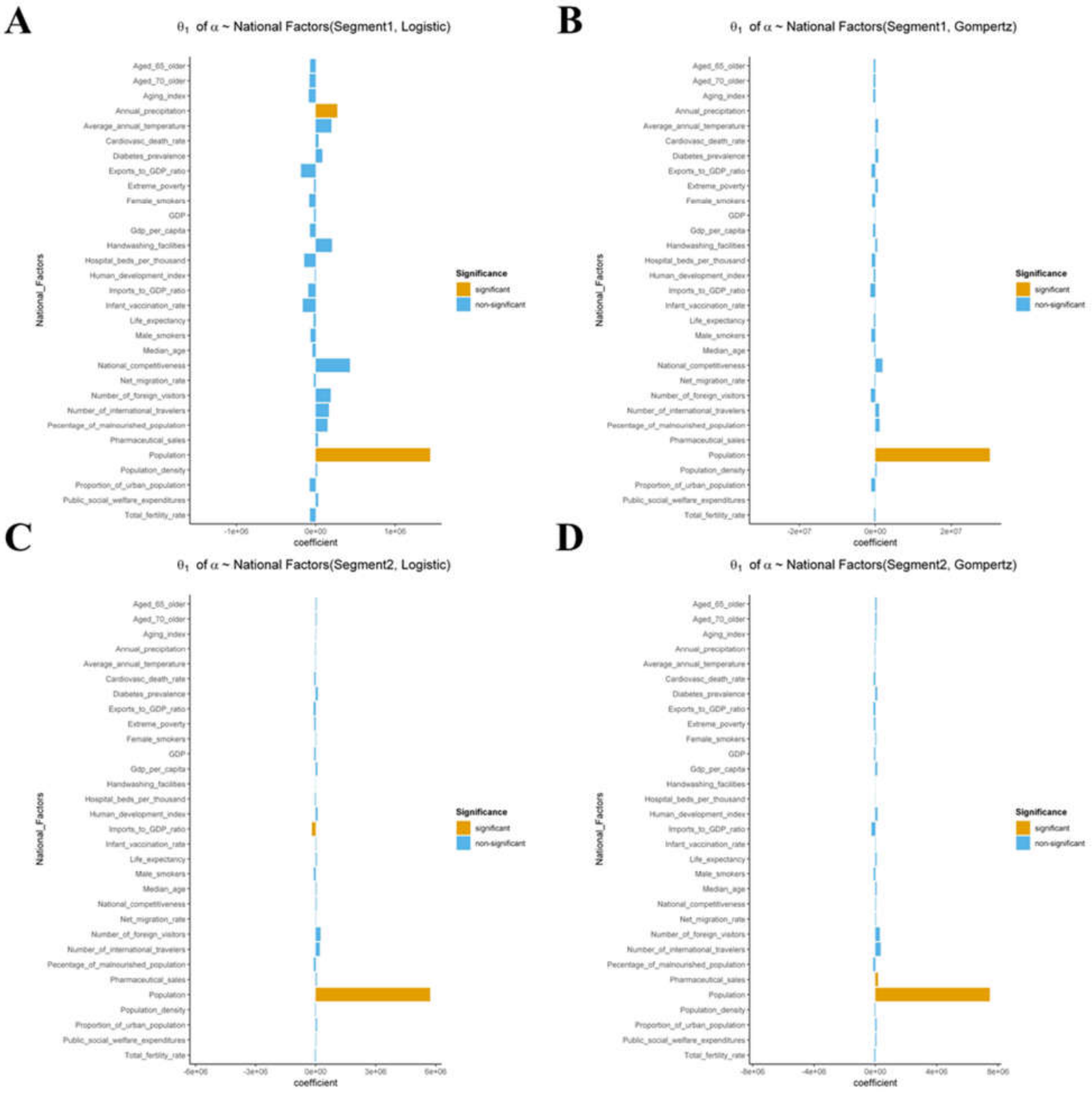

Figure S9. Coefficients of the relationship between national factors and **α**.

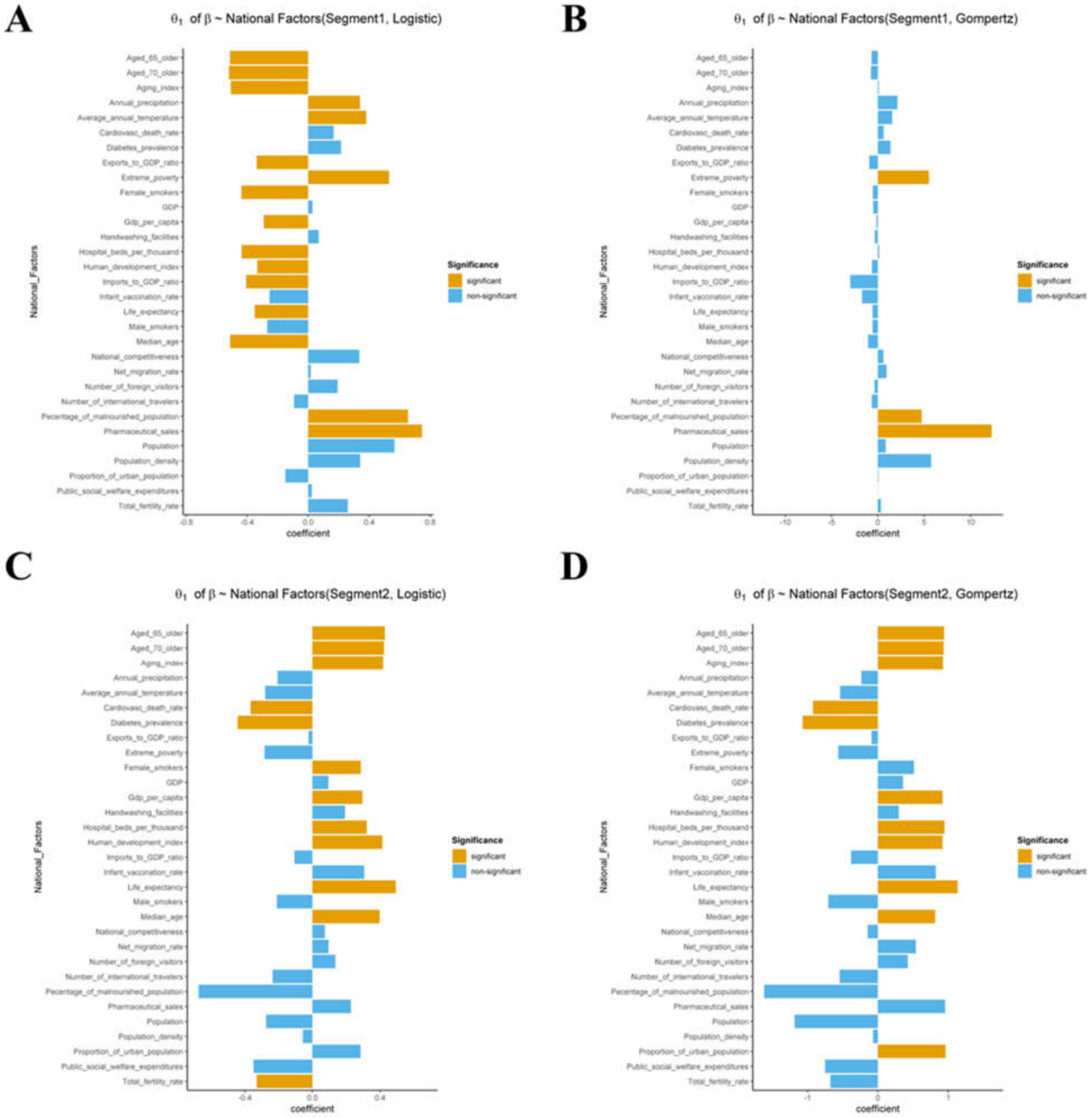

Figure S10. Coefficients of the relationship between national factors and **β**.

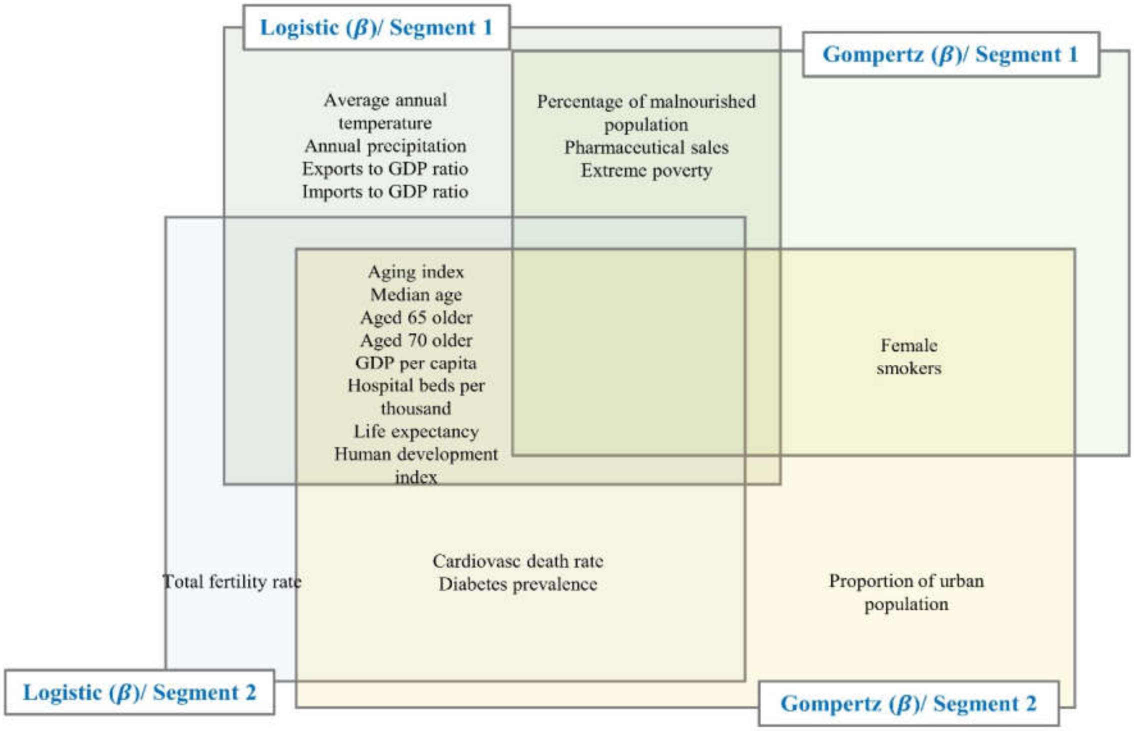

Figure S11. Significant national factors with **β**
